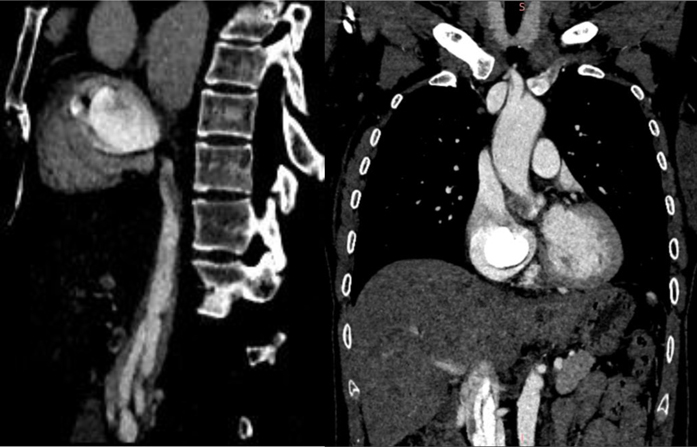# Chest tightness caused by intravenous leiomyomatosis

**DOI:** 10.1016/j.jvsv.2024.101909

**Published:** 2024-05-21

**Authors:** Yong Xie, Tian-Shi Lyu, Li Song, Xiao-Qiang Tong, Jian Wang, Ying-Hua Zou

**Affiliations:** Department of Interventional Radiology and Vascular Surgery, Peking University First Hospital, Beijing, China

**Figure:**
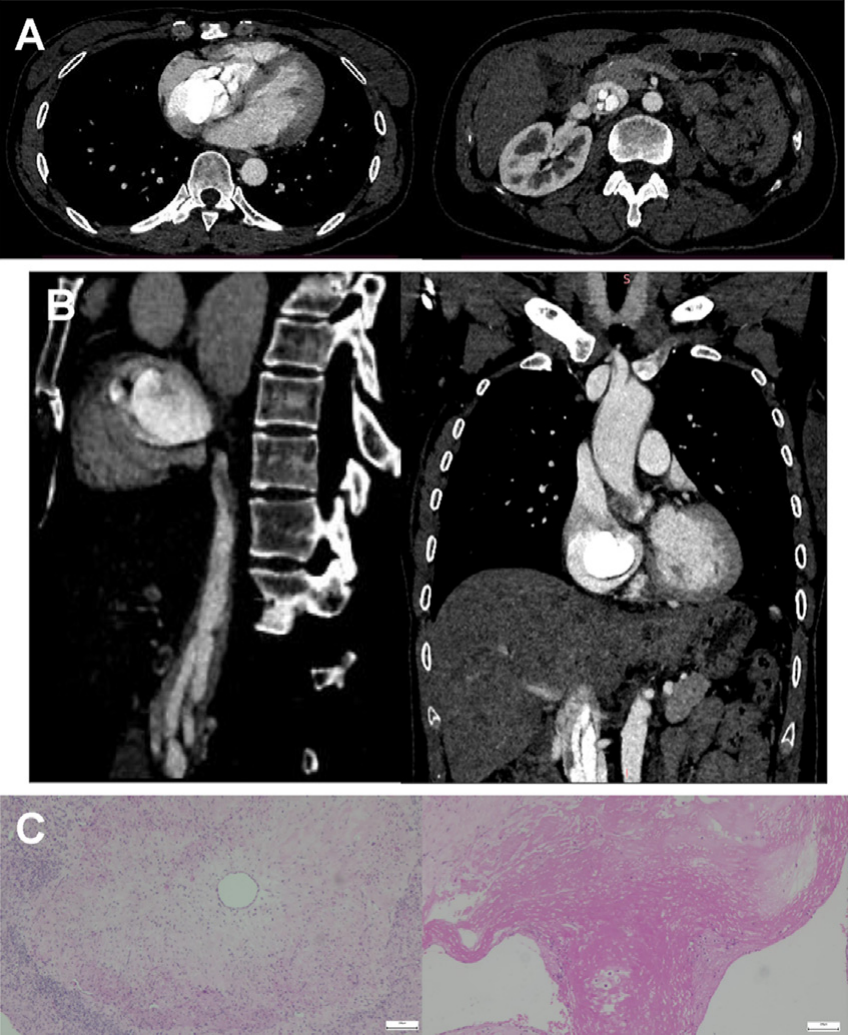

A 49-year-old woman, who provided consent for this publication, presented with chest tightness and shortness of breath for a 1-month duration. The patient's past medical history included uterine fibroids status post myomectomy. Contrast-enhanced computed tomography scan revealed worm-like structures visible in the vena cava, iliac veins, and right side of the heart, suggestive of intravenous leiomyomatosis (IVL) (*A* and *B*/Cover). The patient had no venous thrombotic history or symptoms. A comprehensive surgical procedure involving multiple disciplines, including tumor resection of atrium and the inferior vena cava, as well as artificial vascular diversion of the right external iliac vein/inferior vena cava under general anesthesia was performed for the patient to achieve complete excision of all tumors. Postoperative pathological analysis aided in confirming the diagnosis, with immunohistochemistry showing positivity for smooth muscle actin and desmin. Biopsy of tissue samples showed a significant presence of benign hyperplastic smooth muscle cells within the vein (*C*). The patient's recovery after the surgery was smooth with no complications, and she remained symptom free during the 10-month follow-up period.

IVL is a rare benign leiomyoma typically originating in the pelvic veins and spreading upward along the venous system, affecting the iliac veins, inferior vena cava, and heart.^1^^,^^2^ Characteristic worm-like structures are visibly present in the myometrial, parametrial, or pelvic vessels.^1^ Potential differential diagnoses include right heart tumors (such as atrial myxoma and fibromas), tumor thrombosis of the inferior vena cava, subacute or chronic thrombosis of the inferior vena cava, and primary inferior vena cava leiomyosarcoma. Although the prognosis for IVL is generally benign, surgical intervention with radical tumor removal is considered the most effective treatment to minimize morbidity, mortality, and the likelihood of recurrence.^1^

## Disclosures

None.

## Figures and Tables

**Cover image:**